# ROLE OF COPING STYLES AND NEGATIVE LIFE EVENTS ON HIGHER PERCEIVED MENTAL FATIGABILITY IN OLDER ADULTS

**DOI:** 10.1093/geroni/igz038.866

**Published:** 2019-11-08

**Authors:** Theresa Gmelin, Stacy L Andersen, Robert M Boudreau, Kaare Christensen, Mary K Wojczynski, Stephanie Cosentino, Nancy W Glynn

**Affiliations:** 1 University of Pittsburgh. Department of Epidemiology, Pittsburgh, Pennsylvania, United States; 2 Boston University School of Medicine, Boston, Massachusetts, United States; 3 University of Pittsburgh, Pittsburgh, Pennsylvania, United States; 4 University of Southern Denmark, Odense, Syddanmark, Denmark; 5 Washington University School of Medicine in St. Louis, St Louis, Missouri, United States; 6 Columbia University, New York, New York, United States

## Abstract

Older adults are vulnerable to negative recent life events (RLE) which deplete attentional resources and leads to cognitive exhaustion. Adaptive coping styles reduce perceived stress severity but their role on cognitive tiredness is unknown. We examined RLE and coping styles on perceived mental fatigability (Pittsburgh Fatigability Scale (PFS), 0-50pts, higher=greater fatigability) in the Long Life Family Study (N=1464, age=74.7±12.6, female=57.7%, 43.9% ≥1 major RLE past 6 months, 27.8% higher mental fatigability≥13). All analyses adjusted for family structure, field center, age, and sex. PFS mental scores correlated with all NEO-FFI (60-item, 5-domain) personality traits representing maladaptive (neuroticism r=0.25 p<.0001) and adaptive (conscientiousness r=-0.18, extraversion r=-0.24, p<.00001) coping. Having ≥1RLE was associated with higher mental fatigability (OR=1.4, 95% CI:1.2,1.8, p=.0004); adjustment for neuroticism (OR=1.3, 95% CI:0.9,1.7, p=.06) attenuated the association. Education on adaptive coping may be a modifiable skill that allows older adults to maintain lower perceived mental fatigability despite stressful events.

